# The Possible Link Between Manufacturing and Probiotic Efficacy; a Molecular Point of View on *Bifidobacterium*

**DOI:** 10.3389/fmicb.2021.812536

**Published:** 2021-12-24

**Authors:** Stéphane Duboux, Myrthe Van Wijchen, Michiel Kleerebezem

**Affiliations:** ^1^Nestlé Research, Lausanne, Switzerland; ^2^Host-Microbe Interactomics Group, Wageningen University and Research, Wageningen, Netherlands

**Keywords:** probiotic, niche factors, effector molecules, lactobacillaceae, bifidobacteria, manufacturing

## Abstract

Probiotics for food or supplement use have been studied in numerous clinical trials, addressing a broad variety of diseases, and conditions. However, discrepancies were observed in the clinical outcomes stemming from the use of lactobacillaceae and bifidobacteria strains. These differences are often attributed to variations in the clinical trial protocol like trial design, included target population, probiotic dosage, or outcome parameters measured. However, a contribution of the methods used to produce the live bioactive ingredients should not be neglected as a possible additional factor in the observed clinical outcome variations. It is well established that manufacturing conditions play a role in determining the survival and viability of probiotics, but much less is known about their influence on the probiotic molecular composition and functionality. In this review, we briefly summarize the evidence obtained for *Lacticaseibacillus rhamnosus* GG and *Lactiplantibacillus plantarum* WCFS1, highlighting that expression and presence of probiotic niche factor (NF) and/or effector molecules (EM) may be altered during production of those two well-characterized lactobacillaceae probiotic strains. Subsequently, we summarize in more depth what is the present state of knowledge about bifidobacterial probiotic NF and EM; how their expression may be modified by manufacturing related environmental factors and how that may affect their biological activity in the host. This review highlights the importance of gathering knowledge on probiotic NF and EM, to validate them as surrogate markers of probiotic functionality. We further propose that monitoring of validated NF and/or EM during production and/or in the final preparation could complement viable count assessments that are currently applied in industry. Overall, we suggest that implementation of molecular level quality controls (i.e., based on validated NF and EM), could provide mode of action based *in vitro* tests contributing to better control the health-promoting reliability of probiotic products.

## Introduction

Initially formulated by the World Health Organization in 2002 (Joint Fao/Who Working Group Report on Drafting, 2002) and slightly corrected by experts in the field in 2014 (Hill et al., 2014), probiotics are today defined as “live microorganisms that, when administered in adequate amounts, confer a health benefit to the host.” Overall, probiotic bacteria for food or supplement use (mainly lactobacillaceae and bifidobacteria) have been studied in a large number of clinical trials, targeting a wide array of diseases and conditions (Dronkers et al., 2020).

Two distinguishable classes of health benefits are attributed to probiotics: a “general” class of effects that groups beneficial effects exerted by various well-studied microbial species; and a “strain-specific” class of effects that are expected to be driven by specific probiotics strains. An expert panel convened in 2013 has acknowledged those two classes, concluding that “general” benefits such as “creating a more favorable gut environment” and “supporting a healthy digestive tract” (regrouping a diversity of clinical end points such as diarrhea, antibiotic-associated diarrhea (AAD), gut transit, abdominal pain, bloating, and necrotizing enterocolitis) are displayed by a large number of probiotic strains representing various commonly studied species. The mechanisms of action supporting those “general” probiotic beneficial effects (e.g., probiotic and/or microbiome mediated SCFA production, regulation of intestinal transit, competitive exclusion of pathogens) are similarly believed to be shared by a large number, if not all, of the probiotic strains (Hill et al., 2014). Furthermore, “general” benefits (e.g., AAD prevention) provided by the commonly used *Lacticaseibacillus rhamnosus* GG strain have be shown to be relatively consistent throughout different clinical trials in children (Szajewska and Hojsak, 2020).

In contrast, “strain-specific” benefits are defined as effects that are likely exerted by a limited number of strains, such as “prevention of allergic disease,” “downregulation of inflammation,” “enhancement of anti-infection activities,” or “support of specific organs health” (e.g., reproductive tract, lungs) (Hill et al., 2014). Those beneficial effects are believed to be driven by specific molecules present within or at the surface of the probiotic bacterial cells (Lebeer et al., 2008; Remus et al., 2012; Lee et al., 2013). In the last decade, it was shown that a range of molecules produced by probiotic contribute to their robustness and stress tolerance, supporting their survival and establishment when they transit through the gastro-intestinal tract (i.e., so-called niche factors; NF). In addition, various probiotic effector molecules (EM) have been identified to drive *in situ* host-microbe interactions, thus determining the specific health benefit of different probiotic strains (Lebeer et al., 2018). Disentangling the NF or EM role of specific probiotic molecules is not trivial, especially when adhesive-like phenotypes are affected. Adhesion to the host cells or to the intestinal mucus can be regarded as a factor promoting the bacterial colonization but could as well contribute to the exposure of different structures present on the bacterial cell envelope.

Variability in health effects is not uncommonly observed in clinical trials with probiotic for food or supplement use (Osborn and Sinn, 2007; Johnston et al., 2011; Guo et al., 2019). The inconsistency in results is usually attributed to variations in the design of the clinical studies, including differences in dosage of the probiotic, selection of different target population (i.e., inclusion and exclusion factors at enrollment), powering of the studies according to the primary and secondary objectives, duration of the studies, probiotic delivery format and schedule, data collection, and further analysis performed. Indeed, these factors have been suggested to explain part of the discrepancies observed in the reported clinical health-outcomes (Forssten et al., 2020). Furthermore, probiotics need to exert their effects in the complex microbiome. Inter-individual microbiota variability represent hence a challenge in ensuring probiotic effect consistency in different populations, and new stratification as well as personalized nutrition approaches have been recently proposed to improve the situation (Veiga et al., 2020). Moreover, the way the probiotic strains themselves are produced and formulated is often not well-described in clinical trial studies, while it could play an important role in the health-promoting efficacy that a product elicits. This is well illustrated by the discrepancies observed in randomized clinical trials using *L. rhamnosus* GG targeting the prevention of allergic disease, which are summarized by Segers and Lebeer (2014). Initially, Kalliomaki et al. (2001, 2007) showed in a landmark study that *L. rhamnosus* GG treatment significantly lowered the risk of eczema in young children belonging to families with a history of atopic disease. However, in a subsequent attempt to reproduce this Finnish study protocol, Kopp et al. (2008) failed to detect similar beneficial effects in a German cohort. Population (Finnish vs. Germans) and dosage differences (2^E10^ vs. 1^E10^ CFU daily) are potential confounding factor in those two studies, but it is important to note that the source (and possibly the manufacturing process) of *L. rhamnosus* GG in the studies by Kalliomaki et al. (2001, 2007) and Kopp et al. (2008) was different, which deserves attention because it may have as well contributed to the differences in clinical outcomes (Tripathi et al., 2012). At present, probiotic manufacturing procedures remain largely unexplored as a potential source of variation in probiotic clinical trials outcomes and hence deserves to be studied in more details (Sanders et al., 2014; Brussow, 2019), especially in the light of the increasing knowledge about specific NF and EM that play a role in the efficacy of intestinal delivery and health promotion following consumption of the product.

**FIGURE 1:**
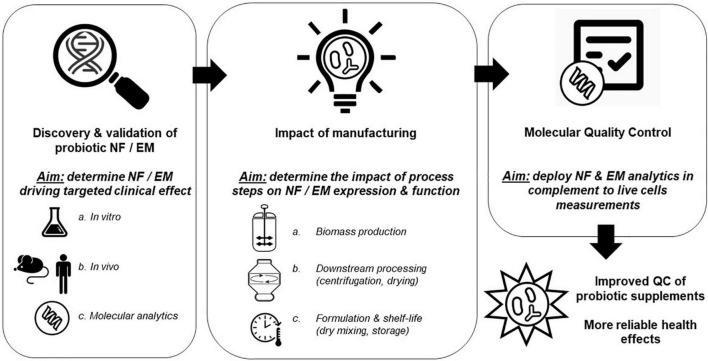
Stepwise approach leveraging probiotic niche factors (NF) and effector molecules (EM) knowledge to improve probiotic supplements consistency. As a first step, a robust link between the presence of probiotic NF or EM and the desired clinical outcome needs to be established. This step will also allow the development of sets of molecular analytics that can be used in the subsequent steps. Then, the different manufacturing steps need to be evaluated to understand their contribution to NF or EM expression and function. Finally, the NF/EM molecular analytics developed earlier can be used as quality control (QC), complementing traditional CFU/live cells measurements, which overall should enable production of probiotic supplements with increased consistency in their attributed health benefits.

Production of dried probiotic supplements consists generally of (a) a series of fermentations of different scale where bacterial biomass is produced using specific media and growth conditions, (b) a centrifugation step to remove the culture supernatant and concentrate the biomass, (c) a mixing step where protectants are added, followed by (d) a drying step (Fenster et al., 2019). Throughout these manufacturing stages, probiotics encounter a range of different stress conditions, including variations in temperature, acid exposure, osmotic and oxidative stress, all of which can modulate their physiology and molecular composition. These modulations may impact their survival during manufacturing as well as their fitness during gastrointestinal tract transit (Corcoran et al., 2008; Gaucher et al., 2019). We suggest that not only the expression of NF (e.g., proteins that contribute to robustness and stress tolerance) can be affected by the production conditions, but also effector molecule expression levels may differ, leading to variable presence of molecules that have been shown to mediate the health-benefit elicited by the strain. The required presence of NF and/or EM is further supported by the fact that probiotic cells rendered metabolically inactive by the mean of heat-treatment can still elicit beneficial health effects (Pique et al., 2019), highlighting the potential limitation of using live cells enumeration alone to ascertain efficacy of probiotic preparations. Therefore, monitoring the expression of validated NF as well as EM during probiotic manufacturing may enable better control of product properties at a molecular level, which goes beyond the traditionally used colony forming units (CFU), and could contribute to an increased robustness of clinical outcomes (Figure 1).

**FIGURE 2:**
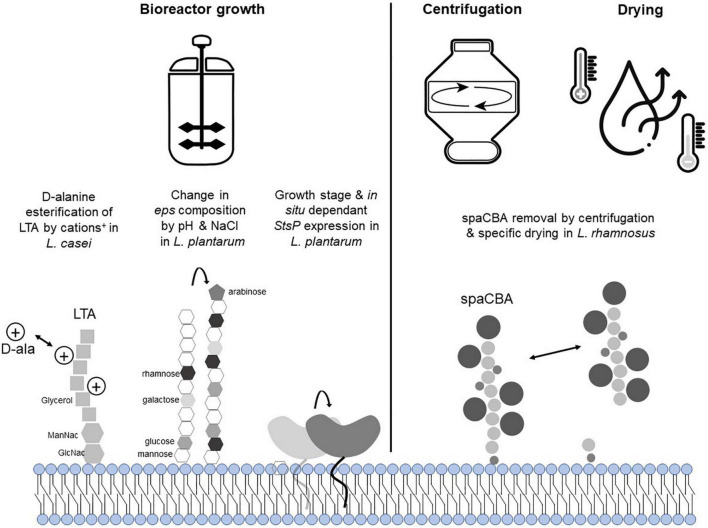
Overview of potential effects of manufacturing on lactobacillacae NF and EM. *L. rhamnosus* GG spaCBA pili has been shown to be at least partially removed by centrifugation (8,000 g, 30 min) or spray-drying. StsP has been shown to be predominantly produced in the stationary growth phase of *L. plantarum* WCFS1, and is regulated in response to intestinal conditions (Bron et al., 2004; Marco et al., 2010; Remus et al., 2012). In analogy to what has been shown in *L. casei* and *L. plantarum*, it can be proposed that lipoteichoic acid (LTA) and exopolysaccharides (EPS) structures of *L. rhamnosus* GG can be modified by cations and/or other positively charged compounds.

In this review, we first briefly highlight that expression and presence of probiotic EM may be altered during production using two well-characterized examples among the lactobacillaceae probiotic, i.e., *Lacticaseibacillus rhamnosus* GG and *Lactiplantibacillus plantarum* WCFS1 (Figure 2). Subsequently, we focus with more depth on bifidobacteria and their probiotic NF and EM, summarizing what is the present state of knowledge about bifidobacterial NF and EM; how their expression may be modified by manufacturing related environmental factors and how that may affect their biological activity in the host (Table 1). Overall, this review highlights the importance of gathering knowledge on probiotic NF and EM, to validate them as surrogate markers of probiotic functionality. We further hypothesize that understanding the dynamics of these molecules during production could contribute to better control the health-promoting reliability of dried probiotic products. Hence, NF/EM based *in vitro* assays represent molecular-level quality controls that adds to the limited information coming from viable count assessments that are currently applied in industry and research.

**TABLE 1:** Summary of known bifidobacterial NF and EM, their validation level and related evidence supporting an effect of manufacturing.

Protein	Bioactivity class and validation level	Evidence of potential manufacturing Impact
Sortase dependent pili	Niche factor; *in vitro* (Turroni et al., 2013)	Growth phase dependent transcription in *B. bifidum* (Westermann et al., 2012)
	Effector molecule; *in vitro* and *in vivo* (Turroni et al., 2013)	Carbohydrate regulated transcription in *B. bifidum* (Foroni et al., 2011; Serafini et al., 2014) and *B. adolescentis* (Duranti et al., 2014)
		Lysine presence is necessary for protein production in *B. bifidum* (Turroni et al., 2014)
Type IVb TAD pili	Niche factor; *in vivo* (O’Connell Motherway et al., 2011)	Growth phase dependent transcription in *B. bifidum* (Westermann et al., 2012)
	Effector molecule; *in vitro* and *in vivo* (O’Connell Motherway et al., 2019)	
Serpin	Niche factor; *in vitro* (Ivanov et al., 2006)	Carbohydrate substrate controls protein presence in *B. longum* (Duboux et al., 2021)
	Effector molecule; *in vivo* (McCarville et al., 2017)	Protease presence controls expression in *B. breve* (Turroni et al., 2010; Alvarez-Martin et al., 2012)
Moonlighting proteins (transaldolase, enolase, DnaK)	Niche factor; *in vitro* (Candela et al., 2007; Gleinser et al., 2012; González-Rodríguez et al., 2012)	Unknown metabolite presence during growth enhance extracellular vehicle production in *B. longum* (Nishiyama et al., 2020)
EPS	Niche factor; *in vitro* (Wu et al., 2010; Xu et al., 2011; Amiri et al., 2019)	Carbohydrate substrate modified transcription (Audy et al., 2010) and yield in *B. longum* (Roberts et al., 1995)
	Effector molecule; *in vitro* (López et al., 2012; Hidalgo-Cantabrana et al., 2013, 2016) and *in vivo* (Xu et al., 2017), but lack structure/function relationship	Growth conditions modulate EPS yield in *B. animalis* (Amiri et al., 2019) and *B. longum* (Ninomiya et al., 2009)

## Modification of *Lacticaseibacillus rhamnosus* GG Effector Molecules by Processing

*L. rhamnosus* GG is among the probiotic strains with the best described set of EM, several of which play diverse roles in the probiotic activity of this strain as assessed mainly in preclinical models. Those include the major secreted proteins p40 and p75 that prevent cytokine-induced inflammatory damage, lipoteichoic acid (LTA) that negatively modulate colitis, CpG-rich DNA motifs that dampen allergen-specific IgE, and exopoly-saccharides (EPS) that reduce adipogenesis in high-fat-diet fed mice (Lebeer et al., 2018). *L. rhamnosus* GG *spaCBA* encoded sortase-dependent pilin anchored at the surface of the bacteria are important NF as they are involved in the mucus and intestinal epithelium adhesion capacity of the strain. However, they were also shown *in vitro* to act as EM as they contribute to the immunomodulatory capacities of the strain when incubated with monocytes and dentritic cells (Lebeer et al., 2012; Vargas Garcia et al., 2015), and stimulate cell proliferation that protects against radiologically induced intestinal epithelial damage (Ardita et al., 2014). Nevertheless, the regulation of expression and functional properties of the pili as well as the other EM during manufacturing of *L. rhamnosus* GG remains largely unexplored.

For example, the regulation of the genes encoding p40 and p75 remains unknown. Additionally, these bioactive molecules are derived from cell wall associated muramidases and are at least partially secreted in the culture supernatant (Yan et al., 2013), which is usually removed during dried probiotic manufacturing (Fenster et al., 2019), raising doubts about their functional availability in supplement products. Similarly, although the role of specific genes involved in LTA biosynthesis in lactobacilli (including *L. rhamnosus* GG) has been studied (Debabov et al., 1996, 2000), their regulation by environmental conditions remains largely unknown. For example, Dlt mediated LTA D-alanylation has been long recognized as an important modulator of the host-effects elicited by LTA (Claes et al., 2012), but we do not know whether *dlt* expression is regulated by environmental conditions in *L. rhamnosus* GG. Notably, it has been demonstrated that the *dlt* gene of *Staphylococcus aureu*s, is regulated by cations levels in the medium [Na^+^, Mg^(2+)^, Ca^(2+)^] (Koprivnjak et al., 2006), and in *L. casei* (a close relative of *L. rhamnosus*) is regulated by the presence of charged molecules like antimicrobial peptides (Revilla-Guarinos et al., 2013). These findings suggest that *dlt* regulation in lactobacilli may be coordinated similarly to what was observed in *S. aureus*, and that the concentrations of positively charged components in the growth medium may affect D-alanylation of LTA. A specific galactose-rich exopolysaccharide in *L. rhamnosus* GG has been previously identified to play a role in the adhesion capacity of the strain (Lebeer et al., 2009). Although regulation of the production of this EPS in *L. rhamnosus* GG has not been studied in detail, recent studies in *L. plantarum* VAL6 indicated that expression of *eps* genes eliciting structural changes of the polysaccharides produced in this species is regulated by pH and sodium chloride induced stress (Nguyen et al., 2021), conditions that may occur during industrial growth. Importantly, manufacturing was demonstrated to influence the presence of the SpaCBA pili at *L. rhamnosus* GG’s surface and could thus affect the presence and function of this important niche factor and effector molecule in preparations of this strain. It has been shown that centrifugation at 8,000 g for 30 min was sufficient to break and separate the pili from the surface of the bacteria (Tripathi et al., 2012), while a specific type of drying (spray-drying without addition of any protectants, which is not a common manufacturing practice) diminished the adherence capacity of *L. rhamnosus* GG correlated with the disappearance of the SpaCBA pili (Kiekens et al., 2019; Figure 2). It is not known today if the presence of this importance protein structure can be influenced by other types of drying. However, freeze-drying was shown to decrease the adherence capacity of *L. rhamnosus* GG, while it did not exert the same effect on *L. casei* Shirota (du Toit et al., 2013). Moreover, besides the physical presence or absence of the pili structure in preparations of this strain, the genetic region encoding the SpaCBA pili was shown to be relatively unstable (Sybesma et al., 2013), which may also contribute to variations in *in vivo* behavior of *L. rhamnosus* GG isolates originating from different products (Grzeskowiak et al., 2011). Altogether, these lines of evidence indicate that upstream (e.g., fermentation conditions) or downstream processing conditions (e.g., centrifugation, type of drying) can play a role in the presence and bioavailability of NF as well as EM of *L. rhamnosus* GG. *In vivo* demonstration of the impact of those processing induced modifications has not yet been pursued, but we hypothesize that they may have contributed to the different outcomes obtained in clinical trials like those reported by Kalliomaki et al. (2001, 2007) and Kopp et al. (2008).

## *Lactiplantibacillus plantarum* WCSF1 Growth Phase Influences Its Host Immunodulatory Capacity

To the best of our knowledge, the only substantiated example demonstrating that production parameters (i.e., growth phase harvesting) can influence the way a probiotic can interact with the human body has been obtained with the well-characterized *L. plantarum* WCFS1 strain. Freeze-dried preparations of heat-killed or live *L. plantarum* WCFS1 were administered to healthy adults. In addition, the live preparations consisted of cells harvested during mid-logarithmic or during the stationary phase of growth. Following consumption of these distinct preparations of the same strain, duodenal tissue biopsies were analyzed by array-based transcriptomics, revealing that both live and dead (heat treated) stationary phase harvested bacterial preparations were able to modulate Nfκ-B responses in human duodenum mucosal tissues, which were interpreted to play an important role in the establishment of immune tolerance. Conversely, the bacteria harvested mid-exponentially failed to induce such responses, but modified the expression of human genes involved in immune-suppressing activities such as BCL3, Iκ-B, and ADM, as well as several functions involved in cell-cycle and metabolic regulation (van Baarlen et al., 2009). As a follow-up, it was found that the *lp_0800* gene, coding for a serine- and threonine-rich surface protein (StsP) that is anchored to the peptidoglycan by sortase was shown to be expressed predominantly during the stationary growth phase, albeit at low levels during growth under laboratory conditions. Notably, previous studies of *L. plantarum* had established that the expression of *lp_0800* was *in situ* induced during the transit through the murine and human intestinal tract, supporting that specific environmental conditions can modulate its expression (Bron et al., 2004; Marco et al., 2007, 2010). Importantly, using isogenic *L. plantarum* WCFS1 lacking or overexpressing StsP, it was shown that StsP surface derived peptides obtained by whole-cell trypsin-shaving could strongly inhibit flagellin induced Nfκ-B activation in a CaCo-2-derived reporter cell line. Finally, gel-purified StsP protein derived tryptic peptides potently suppressed NFkB activation, unambiguously pinpointing this activity to peptides derived from this surface protein (Remus et al., 2012). These findings demonstrate that the growth phase as well as specific growth conditions (i.e., gut-like conditions) of *L. plantarum* WCFS1 can influence the expression level or bioavailability of the important immunomodulatory StsP, which was proposed to play a prominent role in the clinically observed duodenal transcriptional responses (Figure 2).

Of note, the host responses were determined in the duodenum of the participating volunteers. In fact, upon ingestion, the relatively short transit time to reach the duodenal mucosa likely allows a limited molecular adaptation of the probiotic bacteria. At this moment it is unclear whether similar transcriptome response differences would be observed in the colonic mucosa when applying these distinct *L. plantarum* WCFS1 preparations. One the one hand, the different molecular make-up of the preparations may change during gastrointestinal transit, and on the other hand it is known that *stsP* expression is induced in the intestinal tract. We hence hypothesize that ensuring NF and EM presence and function in probiotic products may be especially relevant when the probiotic is expected to elicit its health benefit in the proximal regions of the intestine.

Overall, the *L. plantarum* WCFS1 example strongly indicates that upstream processing (e.g., harvesting time) can impact probiotic bioactivities *in vivo*, and underlines the importance of harvesting probiotic cells at stationary phase, which is today a common practice in industry. Moreover, it supports that quantification of StsP in preparations of *L. plantarum* WCFS1 could serve as a molecular quality control parameter to complement the traditionally used CFU enumeration.

## And What About *Bifidobacterium*?

Similar to probiotics belonging to the *Lactobacillaceae* family, we propose that manufacturing procedures used for bifidobacteria should be investigated for their possible contribution to the discrepancies observed in clinical trial outcomes (Szajewska and Hojsak, 2020). To date, there are only few studies focusing on the effect of manufacturing on bifidobacteria bioactivities and most focused on the potential impact of downstream processing (i.e., drying). Moreover, the available studies did not include an assessment of specific effector molecule presence and bioavailability but were mostly driven by functional assays. For example, Laconelli et al. (2015) showed that different drying procedures (air-, freeze-, spray-drying) impacted on the anti-inflammatory properties of *B. bifidum*, as determined by cytokine production profiling in Peripheral Blood Mononuclear Cells (PBMC) following co-incubation with the differently processed bacterial preparations of the same strain. In addition, this study demonstrated that different down-stream process may affect the hydrophobicity of the strain, which is indicative of changes in cell-surface properties (Laconelli et al., 2015). Conversely, similar analyses showed that spray-drying did not alter the immunomodulatory potential of two *B. animalis* subsp. *lactis* strains (INL1 and BB12), nor did it modify their preventive effect on colitis *in vivo* (Burns et al., 2017). Similarly, although freeze-drying was proposed to enhance the adherence capacity of *B. animalis* subsp. *lactis* BB12, increasing its capacity to outcompete *C. difficile in vitro* (du Toit et al., 2013), these effects were not observed for other strains of *B. animalis* subsp. *lactis* (Charnchai et al., 2016). These studies illustrate the rather limited information concerning the potential impact of manufacturing on bifidobacteria bioactivity, and highlight the contradictory findings described to date on the potential role of downstream processing (e.g., drying) in influencing *Bifidobacterium* probiotic functionality. However, these studies mostly addressed the consequences of different downstream-processing conditions (i.e., drying procedures) on *in vitro* outcomes (O’Connell Motherway et al., 2011; Westermann et al., 2016), whereas the upstream processing (e.g., fermentation parameters) effects on the expression of NF and/or EM in these bacteria remain to be deciphered.

### Metabolic Regulation of Pili Production

Two types of pili have been described in bifidobacteria to act as NF and EM, the sortase dependant pili and the Type IV TAD pili. Sortase dependent pili gene clusters consisting of major (fimA or fimP) and minor (fimB or fimQ) subunit structural proteins are widely distributed amongst *Bifidobacterium* species (Foroni et al., 2011). However, their genetic distribution among strains and species within this genus appears quite disperse. For example, *B. adolescentis* contains five distinct pili encoding gene clusters, while other bifidobacteria, like *B. bifidum*, contains “only” three of these clusters. Out of the three pili gene clusters found in *B. bifidum* PRL2010, only *pil2* and *pil3* were found to be functional, and *pil1* being disrupted by a frameshift (Foroni et al., 2011; Turroni et al., 2013; Duranti et al., 2014). Importantly, and analogous to what was shown for *L. rhamnosus GG*, the sortase dependent pili of *B. bifidum* PRL2010 were demonstrated to play a role in both adhesive and anti-inflammatory properties of the strain using recombinant *L. lactis* harboring the *pil2* or *pil3* gene clusters. While Pil2 was shown to act as a NF and mediated binding to extracellular matrix, Pil3 was also able to modify both *in vitro* and *in vivo* inflammatory responses (Turroni et al., 2013).

Similarly to the sortase dependent pili, type IVb TAD pili are also conserved and widely distributed in both gram positive and gram negative bacteria (Pu et al., 2018), and has been identified in multiple *B. breve* and *B. bifidum* strains (O’Connell Motherway et al., 2011; Westermann et al., 2012). It was demonstrated that the Type IVb TAD pili of *B. breve* UCC2003 act as NF, as disruption of the ATPase encoding gene *tadA*_2003_, which is essential for its assembly, resulted in a decreased capacity of the strain to colonize the mouse intestine (O’Connell Motherway et al., 2011). Additionally, using a set of recombinant *B. breve* UCC 2003 strains it was shown that the same pilin structure (and particularly its TadE pilin subunit) could contribute to the maturation of the naïve gut, since it promoted epithelial proliferation both *in vitro* and *in vivo* (O’Connell Motherway et al., 2019), demonstrating its additional EM role.

Limited information is available about the regulation of production of the various pili that are encoded by bifidobacteria. In *B. bifidum* S17 it was demonstrated that sortase-dependent pili encoded by the *pil2* and *pil3* clusters were higher expressed during exponential growth as compared to the stationary phase of growth (Westermann et al., 2012). In another strain of the same species, *B. bifidum* PRL2010, the *pil2* and *pil3* clusters were transcribed both during growth in laboratory medium (MRS) as well as in the mouse cecum, whereas transcription of the *pil1* cluster could not be detected under either of these conditions (Turroni et al., 2013). Culturing of this strain in bovine milk led to activation transcription of the *pil1* cluster genes, indicating that growth (i.e., production) conditions can influence the repertoire of pilin produced by *B. bifidum* PRL2010. This observation was expanded by demonstrating that the other *pil* clusters in this strain were subject to substrate regulation, which is exemplified by the induction of transcription of the *pil2* cluster during growth on fructo-oligosaccharides (FOS) and the induction of the *pil3* cluster during both growth on bovine milk or polysaccharides derived from kefir (Foroni et al., 2011; Serafini et al., 2014). Analogously, the expression of *pil* gene clusters was also regulated by the carbon source used for growth in *B. adolescentis* 22L, where maltodextrin or cellobiose as substrates for growth resulted in an increase of gene expression (compared to growth on glucose) for *pil3*, *pil4*, and *pil5*, which coincided with increased adhesion of the strain to laminin, fibrinogen, and fibronectin, albeit that direct relatedness of these observations remains to be established (Duranti et al., 2014). Besides the carbon source for growth, also the available nitrogen source in the medium has been reported to control pilin expression. For example, the presence of lysine in the growth medium appeared to be essential for Pil2 and Pil3 production by *B. bifidum* PRL2010 (Turroni et al., 2014).

In both *B. breve* and *B. bifidum*, part of the genes encoding the Type IVb TAD pili were found to be expressed during standard growth conditions in the laboratory (O’Connell Motherway et al., 2011; Westermann et al., 2012). However, the transcriptional levels of the Type IVb TAD pilus encoding genes in *B. breve* were strongly induced (25–62-fold) when the bacteria were inhabiting the murine intestinal tract. This was further supported by immunogold staining demonstrating that the pili structures could only be observed when the strain was harvested from the murine gut (O’Connell Motherway et al., 2011). Even though the Type IVb TAD pili protein presence was not assessed in laboratory-grown (MRS) *B. bifidum* S17, the encoding genes (*tadZ, tadA*, and *tadB*) were expressed in a growth phase dependent manner, with higher transcriptional levels in the exponential phase compared to the stationary phase (Westermann et al., 2012).

Although, the specific environmental factors that regulate pili production of specific bifidobacterial pili are quite diverse (e.g., carbon source, nitrogen source, “intestinal conditions,” etc.) and/or remain unknown, it is clear from the observations presented above that pilin expression by *Bifidobacterium* probiotics may be modulated by the growth conditions (e.g., substrate) employed during production. In addition, more work deserves to be pursued to decipher the role of downstream processing, as analogous to what has been described for the pili of *L. rhamnosus* GG, we can hypothesis that the presence of the pili on the cell-surface of the bifidobacteria may be impacted by drying procedures.

### Environmental Factors Regulating Serine Protease Inhibitor Production

The serine protease inhibitor (serpin) of pancreatic and neutrophilic elastases was has initially described in *B. longum* NCC 2705 (Ivanov et al., 2006). This protein was shown to be conserved in a broad range of bifidobacteria and has been proposed to protect them against host produced proteases, thus providing them with a survival and colonization advantage (Ivanov et al., 2006; Turroni et al., 2010). The serpin’s capacity to inhibit the Human Neutrophil Elastase (Ivanov et al., 2006) may also be involved in the immunomodulatory capacities of the strain (Riedel et al., 2006) as elastase is released by activated neutrophils at the sites of intestinal inflammation in the gastro-intestinal tract (Burg and Pillinger, 2001). In line with this role in dampening innate immunity, serpin was demonstrated to play a key role in the anti-inflammatory effect of *B. longum* NCC 2705 in a mouse model of gluten sensitivity (McCarville et al., 2017). In addition, it was recently reported that the serpin of the NCC 2705 strain prevented enteric nerve activation *in vitro*, which was proposed to potentially play a role in pain reduction in Irritable Bowel Syndrome (IBS) patients (Buhner et al., 2018). These findings indicate that analogous to the bifidobacterial pili, the role of the serpin is dualistic in the sense that it acts as both NF and EM.

Transcriptional regulation studies of the *B. breve* UCC2003 serpin-encoding gene showed that it involves a protease inducible two-component system encoded directly adjacent to the serpin encoding operon, which was shown to activate serpin production upon exposure of the strain to proteases (e.g., papain) (Alvarez-Martin et al., 2012). However, a similar two-component system appears to be absent in *B. longum* subsp. *longum* strains (including NCC 2705), and variable gene-syntenies encountered in the serpin encoded region in different bifidobacterial (sub-)species suggests that serpin regulation may involve (sub-)species specific mechanisms (Turroni et al., 2010). This notion is further confirmed by our recent study that demonstrated that in *B. longum* subsp. *longum*, serpin production is regulated by the carbohydrate-substrates used for growth, revealing galactose and fructose (or galacto- or fructo- di/oligo-saccharides) as inducing substrates, while the presence of glucose repressed serpin production almost completely (Duboux et al., 2021). These studies illustrate the diverse environmental factors and regulatory mechanisms involved in controlling serpin production in *Bifidobacterium* (sub-) species, indicating that growth conditions (e.g., substrate) could be tailored to ensure, or even enhance the production and function of this NF and/or EM in the final probiotic preparation.

### Bifidobacterial Exopolysaccharides Biosynthesis and Potential Link to Bioactivities

Exopolysaccharides (EPS) are extracellular carbohydrates polymers synthesized by a vast variety of micro-organisms, including gram positive bacteria. In bifidobacteria, EPS can be covalently or non-covalently bound to the cell surface (sometimes referred to as capsular polysaccharides; CPS), or can be predominantly secreted. The EPS produced by bifidobacteria are heteropolysaccharides (HePS) that have been reported to vary in molecular weight (between 4.9 × 10^3^ and 3 × 10^6^ Da) (Leivers et al., 2011) and monosaccharide composition and linkage (Hidalgo-Cantabrana et al., 2012). The synthesis of HePS by *Bifidobacterium* strains involves gene clusters (*eps* clusters) and biosynthesis mechanisms that are similar to those described for other microbes, involving a membrane associated synthesis machinery that utilizes cytoplasmic sugar nucleotides as building blocks for the assembly of repeating units that are exported and polymerized to form the HePS (Altmann et al., 2016; Schiavi et al., 2016; Castro-Bravo et al., 2017). Most of the bifidobacterial HePS are predominantly composed of D-galactose and D-glucose, but can also contain other monosaccharides like L-rhamnose, D-mannose, L-arabinose, or D-fructose in ratios that can vary between species and likely also between strains of the same species (Xu et al., 2011; Amiri et al., 2019). This notion is supported by the fact that *eps* gene clusters are highly variable between different species and strains of bifidobacteria (Hidalgo-Cantabrana et al., 2014; Altmann et al., 2016; Schiavi et al., 2016; Castro-Bravo et al., 2017).

Purified EPS produced by *B. longum* BCRC14634 showed an anti-microbial effect on four pathogenic and three food spoiling bacteria (Wu et al., 2010), which could provide a competitive advantage to the strain in the complex gut ecosystem, supporting its role as NF. In addition, bifidobacterial EPS has also been proposed to affect host responses, suggesting that these molecules may also act as EM in bifidobacteria. Firstly, EPS produced by two *B. animalis* strains (*B. animalis* RH and *B. animalis* subsp. *lactis* BB12) was shown to possess anti-oxidant capacities *in vitro* (Xu et al., 2011; Amiri et al., 2019), which could be relevant in order to alleviate intestinal oxidative damages. Then, the EPS produced by different *B. longum* and *B. animalis* strains has been proposed to modulate the immune response of the host based on the role of these molecules in inducing immune cell proliferation and modulating cytokine production in peripheral blood mononuclear cells (PBMCs) (López et al., 2012; Xu et al., 2017). Notably, the immunomodulatory effect of EPS observed in this study was shown to be strain specific, which was exemplified by the finding that out of eight strains of *B. longum* and *B. animalis* tested, only the EPS produced by *B. animalis* A1 and *B. longum* NB667 elicited a significant increase of PBMC proliferation (López et al., 2012). The immunomodulatory capacities of specific bifidobacterial EPS molecules has been reported to be quite diverse, including reports on the induction of pro-inflammatory profiles *in vitro* (López et al., 2012; Hidalgo-Cantabrana et al., 2016) or *in vivo* (Xu et al., 2017) but also cases where anti-inflammatory responses were detected (Wu et al., 2010; Schiavi et al., 2016). However, these studies provided very limited information on the physical and chemical characteristics or the monosaccharide composition of the EPS molecules produced by these different bifidobacterial strains, leaving the relationships of EPS structure and its immunomodulatory function unaddressed. A study by Hidalgo-Cantabrana et al. (2013) partly elucidated how EPS characteristics might affect its biological activity. In this study they used *B. animalis* subsp. *lactis* A1 and two mutant derivatives (A1dO and A1dOxR) that produce EPS with distinct monosaccharide composition and molecular size characteristics compared to the wild-type strain. Strain A1dOxR harbored a mutation in the *Balat_1410* tyrosine kinase encoding gene (Hidalgo-Cantabrana et al., 2015) and expressed the enzyme dTDP-glucose 4,6-dehydratase that catalyzes the production of dTDP-rhamnose at an elevated level, leading to the production of a high molecular weight, rhamnose-rich EPS. The changes in polymer length and monosaccharide composition in this mutant strain were associated with increased production of the anti-inflammatory cytokine IL-10 by PBMCs exposed to the A1dOxR strain relative to its parental strain (Hidalgo-Cantabrana et al., 2013). To the best of our knowledge this is one of the few studies where the EPS structure-function relationship is investigated in the context of immunomodulatory capacities, illustrating that there is a large gap in our mechanistic understanding of the postulated role of EPS as EM in bifidobacteria.

Several studies have demonstrated that growth conditions modulate both the level of production as well as the structural properties of EPS produced by bifidobacteria. As an example, the carbon substrate applied during growth influences the expression of *eps* related genes in *B. longum* CRC002 (Audy et al., 2010), which is potentially influencing the EPS produced by the strain. Indeed, the carbon source used for growth of *B. longum* BB79 affected the level of EPS produced, with lactose leading to the highest level of production when compared to glucose, fructose, or sucrose (Roberts et al., 1995). Besides the influence of the carbon source, differences in concentration of yeast extract, growth temperature and incubation time modulated the EPS production by *B. animalis* BB12 (Amiri et al., 2019), and the level of dissolved oxygen and CO2 concentrations affected EPS production in *B. longum* JBL05 (Ninomiya et al., 2009). Although these studies did not investigate the potential compositional changes in the EPS that was produced, they do highlight that various growth conditions can influence EPS production. In this context, it should be noted that in *L. rhamnosus* E/N, the carbon source used for growth did not only affect the quantity of EPS produced but also its monosaccharide composition (Berecka et al., 2013). Taken together, the existing information illustrates the limited and scattered knowledge of the regulatory mechanisms underlying the production of (different) EPS molecules in bifidobacteria. Especially when the role of EPS as a NF or EM is to be further substantiated, better understanding of the regulation of EPS production and composition will be required to reliably investigate the role played by these molecules in *Bifidobacterium* probiotics. Such knowledge would also be required to design manufacturing procedures that aim to improve the presence and abundance of bioactive EPS molecules in *Bifidobacterium* probiotic products, in order to enhance their health benefit reliability as we propose in this review.

### Environmental Regulation of Surface Enzymes Involved in Adhesion

Different cytoplasmic enzymes are also found on the surface of bacterial cells, such as transaldolase, enolase, and DnaK. These surface attached proteins were suggested to act as bifidobacterial NF, based on their *in vitro* demonstrated role in the adhesive properties of *Bifidobacterium* strains. The example proteins mentioned serve typical cytoplasmic functions in glycolysis (transaldolase and enolase) or stress response (DnaK is a chaperonin), but were suggested to be secreted via a yet unknown non-classical secretion mechanism (González-Rodríguez et al., 2012). This class of surface exposed cytoplasmic proteins is often referred to as moonlighting proteins (Candela et al., 2007; Gleinser et al., 2012) defined by the fact that they can perform two or more physiologically relevant biochemical or biophysical functions (Jeffery, 1999). It could be that these proteins become deposited on the cell surface upon lysis of surrounding bacteria that release their cytoplasmic content in the environment. Alternatively, it was recently proposed that in *B. longum* NCC 2705 those type of surface exposed cytoplasmic (moonlighting) proteins could be excreted through the formation of extracellular vesicles (Nishiyama et al., 2020), of which the formation was shown to occur through membrane bubbling upon peptidoglycan damage in *Bacillus subtilis* (Toyofuku et al., 2017).

Modulation of the expression of these cytoplasmic proteins could also affect their surface exposure levels. Although such relation has to the best of our knowledge not been investigated in detail, it has been reported that different environmental factor (pH, bile salts) or mild stress conditions (Sánchez et al., 2007; Candela et al., 2010) can increase expression levels of moonlighting proteins. Finally, extracellular vesicles are one of the purported export-mechanism for adhesive proteins and may be modulated during growth as the presence of yet unidentified gut microbiota derived metabolites (in *in vitro* fecal fermentations) increased their formation (Nishiyama et al., 2020). More work is required to understand how these proteins are ending up on the cell surface, and if different manufacturing steps (e.g., steps inducing lysis of cells or growth condition inducing extracellular vesicles formation) might influence this process.

## Conclusion and Discussion

Discrepancies in clinical trial outcomes stemming from the use of probiotics belonging to the *Lactobacillaceae* family or the *Bifidobacterium* genus has been previously reported. Variations in trial design (population, dose, outcome measurement, etc.) have been advocated to at least in part explain the differences in the outcomes. However, the information summarized in the present review indicates that manufacturing conditions can influence on the presence and/or function of probiotic molecules that play critical roles in the survival of the bacteria in the intestinal tract (NF) and/or their interaction with host cells (EM), which may in turn be an additional cause for the observed variations in clinical outcomes. This review argues that knowledge of probiotic NF and/or EM molecules can provide means to assess the impact of manufacturing conditions on the functionality of the studied strain. Ensuring the presence of validated NF and EM in the final probiotic product could ultimately contribute to improve the consistency of the probiotic’s clinical effect.

As mentioned above, ensuring the presence and function of NF and EM during manufacturing could be particularly relevant for upper-gastrointestinal tract mediated health-benefits. An interesting example supporting this hypothesis is the serpin produced by *B. longum* NCC 2705 that was shown to reduce gliadin-induced immune responses in a mouse model, which was proposed to compensate the duodenal serine protease inhibitor decrease observed in active celiac disease. Recently, it was shown that the level of serpin production by this strain can be strongly modulated by the carbon source applied for growth, which allows the production of serpin-rich and serpin-poor probiotic preparations that can subsequently be evaluated *in vivo*. Such approach could establish the relevance of EM presence and function in the probiotic product for health benefits elicited in the proximal region of the intestine. Conversely, the Type IVb TAD pili of *B. breve* UCC 2003 was demonstrated to act as EM in the promotion of colonic epithelial cell proliferation. In this case, it would be interesting to study if the presence or absence of those EM in the initial preparation influences their mediated effect in the distal regions of the intestine, as pili expression was shown to be induced during intestinal transit.

Overall, this review highlights that beyond an improvement of the current clinical trial designs, there is a need to better understand the impact of manufacturing on clinical efficacy of probiotic products. First, NF and EM molecules have to be established as surrogate markers for probiotic functionality, linking their presence or absence to functional readouts. Work around this topic is relatively advanced for *Lactobacillaceae* (e.g., with the cases of *L. plantarum* WCFS1 and *L. rhamnosus* GG). However, for *Bifidobacterium* probiotics, only a few molecules were identified to act as EM in preclinical animal studies, like the sortase dependant pili of *B. bifidum* PRL2010, the Type IVb TAD pili of *B. breve* UCC 2003 and the serpin of *B. longum* NCC 2705. Overall, additional work, including *in vivo* demonstrations, is needed to identify and validate the molecules driving the host-bifidobacteria interactions. Once NF or EM established as surrogate markers of functionality, implementation of molecular level quality controls (i.e., based on NF and EM), could nicely complement the traditional live cells (e.g., CFU) enumeration in the final probiotic preparation, hence providing more insight on the functional consistency of dried probiotics.

## Author Contributions

SD took the lead in writing the manuscript, under direct supervision of MK. MV provided initial literature survey for several sections, under supervision of SD. All authors provided critical feedback and helped shape the manuscript.

## Conflict of Interest

SD and MV performed the work as employees of Nestlé (Société des Produits Nestlé SA). The remaining author declares that the research was conducted in the absence of any commercial or financial relationships that could be construed as a potential conflict of interest.

## Publisher’s Note

All claims expressed in this article are solely those of the authors and do not necessarily represent those of their affiliated organizations, or those of the publisher, the editors and the reviewers. Any product that may be evaluated in this article, or claim that may be made by its manufacturer, is not guaranteed or endorsed by the publisher.
